# Supplementary material for: Inferring on Speleomantes Foraging Behavior from Gut Contents Examination
Source: Animals (Basel). 2023 Aug 31;13(17):2782. doi: 10.3390/ani13172782 (PMC10486601; doi:10.3390/ani13172782)
Supplement: Supplementary file 1 [file animals-13-02782-s001.zip › animals-2573458-supplementary.pdf]

Table S1. Dataset gathering information on *Speleomantes* gut contents analyzed in this study.

Prey category	Number of recognized items	Percentage related to the total number of recognized prey items
Pulmonata	120	0.68
Sarcoptiformes	210	1.19
Mesostigmata	38	0.22
Trombidiformes	33	0.19
Araneae	684	3.88
Pseudoscorpiones	260	1.47
Opiliones	79	0.45
Lithobiomorpha	41	0.23
Geophilomorpha	26	0.15
Scolopendromorpha	8	0.05
Julida	83	0.47
Glomerida	10	0.06
Polydesmida	234	1.33
Isopoda	242	1.37
Symphypleona	638	3.62
Poduromorpha	52	0.29
Entomobryomorpha	701	3.98
Zygentoma	4	0.02
Ephemeroptera	1	0.01
Odonata_ninfa	1	0.01
Orthoptera	41	0.23
Blattodea	21	0.12
Psocodea	17	0.10
Hemiptera	408	2.31
Endopterygota_larva	1	0.01
Hymenoptera	885	5.02
Hymenoptera_Formicidae	385	2.18
Coleoptera	1247	7.07
Coleoptera_Staphylinidae	1026	5.82
Coleoptera_larva	97	0.55
Neuroptera	2	0.01
Mecoptera	2	0.01
Trichoptera	29	0.16
Plecoptera	192	1.09
Lepidoptera	29	0.16
Lepidoptera_larva	73	0.41

Diptera	9112	51.68
Diptera_larva	512	2.90
Archaeognatha	26	0.15
Tricladida	1	0.01
Gordea	2	0.01
Nematoda	10	0.06
Haplotaxida	37	0.21
Siphonaptera	3	0.02
Dermaptera	5	0.03
Ixodida	2	0.01
